# Our Journal

**Published:** 1877-11-20

**Authors:** 


OUR JOURNAL.—The next number of our Journal will close the volume for 1877. We have labored hard to make THE RECORD interesting and useful to the busy practitioner. That our labors have not been in vain, we have abundant testimony in the increase of our circulation, and the numerous complimentary expressions to be found in the correspondence of the office. Our volume for 1878 will contain more reading matter and will be gotten up more neatly than heretofore, and at the same low rate of subscription. Its practical character will be rigidly maintained, and renewed efforts will be made to present our readers with the pith and cream of all that is useful, and valuable in the home and foreign Journals, together with practical items and formulæ, from intelligent Medical men in all sections of our country.

The necessity for a cheap practical Journal of this character, in our section of the Union must be cle rly apparent to every sensible Medical man; and yet there is a strange and unaccountable indifference among Medical men in the *South*, toward the Journals of their own section, and many will be surprised to learn that nearly half the additions to our list during the prerent year, have been from the extreme Western and Northern States. While we are glad to welcome subscriptions from any direction, yet, we feel that we have a right to expect that the Physicians of Georgia, and the adjacent States should take a special interest in, and work for their own Journal. Once again then we ask, each one of our present subscribers to help us. Renew your subscription, for the coming year, and send us one or more new subscribers, and thus aid us in the important and useful work of promoting Medical literature in the South.

